# Temperature-induced aerobic scope and Hsp70 expression in the sea cucumber *Holothuria scabra*

**DOI:** 10.1371/journal.pone.0214373

**Published:** 2019-03-22

**Authors:** Holger Kühnhold, Nuri Steinmann, Yi-Hsuan Huang, Lisa Indriana, Achim Meyer, Andreas Kunzmann

**Affiliations:** 1 Department of Ecology, Leibniz Centre for Tropical Marine Research (ZMT), Bremen, Germany; 2 Research Centre for Oceanography, Indonesian Institute of Science (LIPI), Lombok, Indonesia; University of Connecticut, UNITED STATES

## Abstract

The Aerobic Scope (AS), which reflects the functional capacity for biological fitness, is a highly relevant proxy to determine thermal tolerance in various taxa. Despite the importance of this method, its implementation is often hindered, due to lacking techniques to accurately measure standard- (SMR) and maximal- (MMR) metabolic rates, especially in sluggish marine invertebrates with low oxygen consumption rates, such as sea cucumbers. In this study the AS concept was modified to define a Temperature-induced Aerobic Scope (TAS), based on metabolic rate changes due to temperature adjustments rather than traditionally used physical activity patterns. Consequentially, temperature dependent peak and bottom O_2_ consumption rates, defined as Temperature-induced Maximal- (TMMR) and Standard Metabolic Rates (TSMR), respectively, served as MMR and SMR alternatives for the sea cucumber *Holothuria scabra*. TMMR and TSMR were induced through acute temperature change (2°C per hour; 17–41°C) until critical warm (WT_crit_) and cold (CT_crit_) temperatures were reached, respectively. In addition, Hsp70 gene expression linked to respiration rates served as synergistic markers to confirm critical threshold temperatures. O_2_ consumption of *H*. *scabra* peaked distinctly at WT_crit_ of 38°C (TMMR = 33.2 ± 4.7 μgO_2_ g^-1^ h^-1^). A clear metabolic bottom line was reached at CT_crit_ of 22°C (TSMR = 2.2 ± 1.4 μgO_2_ g^-1^ h^-1^). Within the thermal window of 22–38°C *H*. *scabra* sustained positive aerobic capacity, with assumed optimal performance range between 29–31.5°C (13.85–18.7 μgO_2_ g^-1^ h^-1^). Between 39–41°C *H*. *scabra* decreased respiration progressively, while gene expression levels of Hsp70 increased significantly at 41°C, indicating prioritization of heat shock response (HSR) and homeostatic disruption. At the cold end (17–22°C) homeostatic disruption was visible through incrementally increasing energetic expenses to fuel basal maintenance costs, but no Hsp70 overexpression occurred. TMMR, TSMR and TAS proved to be reliable metrics, similar to the traditional energetic key parameters MMR, SMR and AS, to determine a specific aerobic performance window for the sluggish bottom dwelling species *H*. *scabra*. In addition, the linkage between respiration physiology and molecular defense mechanisms showed valuable analytical synergies in terms of mechanistic prioritization as response to thermal stress. Overall, this study will help to define lethal temperatures for aquaculture and to predict the effects of environmental stress, such as ocean warming, in *H*. *scabra*.

## Introduction

The determination of species-specific critical temperature limits is highly relevant to predict stress levels due to global environmental change. The biogeography of marine species conforms closely to optimal temperature windows, especially near equatorial boundaries [1, 2]. Tropical species are confined to narrower thermal niches than temperate species, which makes them more susceptible to niche shifts, due to higher metabolic rate changes [3, 4]. A gradual temperature increase of only 2–3°C caused already significant reductions of thermal tolerance levels in various marine invertebrates [5], and substantially decreased aerobic performance [6] as well as compromised growth and reproduction for a number of coral reef fish [7, 8]. Consequently, the effects of global climate change–i.e. a temperature increase of 2–4°C for the tropical oceans–projected to occur by the end of this century, might pose a serious metabolic challenge for many equatorial marine species.

A comprehensive mechanistic explanation for temperature driven effects on organism performance is provided by the oxygen- and capacity- limited thermal tolerance (OCLTT) [9–11]. OCLTT defines physiological capacity limits based on aerobic performance, encompassing the span for oxygen uptake, transport and delivery, the so-called aerobic scope (AS). AS is defined as the difference between maximal performance, known as the maximal metabolic rate (MMR), and resting metabolism, known as the standard metabolic rate (SMR) [12, 13]. The difference between SMR and MMR represents the accessible respiration energy, which is not required for basal maintenance functions and, thus, freely available for functions related to biological fitness (activity, feeding, and reproduction). Within an optimal temperature range the aerobic scope peaks, which correspond to optimal species performance. Above or below this optimal range, performance declines with a narrowing aerobic scope. The aerobic scope is delimited by a species-specific lower and upper critical temperature (T_crit_) at which SMR exceeds MMR and animals depend progressively on unsustainable anaerobic energy supply to fuel physiological functions [11]. At temperature stress beyond T_crit_, extreme hypoxia will lead to terminal ATP deficiency and ultimately cause the loss of vital body functions (i.e. CT_max_) [10, 14]. The aerobic scope has been widely used as a model to determine thermal tolerances for various fish species from temperate [15–17] and tropical [6, 7, 18] regions. For slow-moving benthic marine species such as sea cucumbers, the lack of methods to reliably quantify SMR and MMR largely hinders the assessment of AS. In more mobile organisms (i.e. fish) the temperature effect on the aerobic scope is generally determined by measuring SMR and MMR during lowest (i.e. resting/sleeping) and highest (i.e. fast swimming) physical activity levels, respectively, at different temperatures. In sluggish benthic species such as sea cucumbers, however, the measurement of SMR and MMR through aerobic adjustments as response to induced physical activity challenges is not convenient. Hence, in this study we tested acute temperature challenge itself for the induction of an aerobic bottom line and peak performance in the tropical sea cucumber *Holothuria scabra*, to define metrics similar to the established SMR and MMR.

Besides aerobic performance, critical temperature stress can also be measured at the molecular level through the differential expression of genes encoding heat shock proteins (HSPs) [19, 20]. HSPs act as molecular chaperones and regulate protein folding [21]. Hsp70 has been detected in complexes with proteins presumably assisting in the recovery from stress by repair of damaged proteins [22]. Despite its relevance in thermal stress detection, detailed knowledge on temperature dependent Hsp70 gene expression is still scarce for many marine invertebrates, especially for species without a sequenced and annotated genome, such as *H*. *scabra*. Moreover, knowledge about the direct link between the allocation and prioritization of energy resources to fuel cellular- and tissue- protection, such as Hsp70 synthesis, and variations in aerobic scope is scarce.

*H*. *scabra* contributes significantly to the recycling of nutrients and minerals through bioturbation [23]. Moreover, this species serves as prey for many taxa and enhances biodiversity by forming various symbiotic relations. Besides their ecological relevance, *H*. *scabra* is a high valued aquaculture candidate species [24–26]. Due to its high commercial and ecological relevance, fine-tuned methods to detect thermal stress in *H*. *scabra* are crucial to enhance aquaculture production and to assess this animal’s susceptibility to ocean warming.

The aim of this study was two-fold: 1) To determine temperature-induced maximal and minimal aerobic performance and the corresponding limiting temperatures by measuring O_2_ consumption in *H*. *scabra* along a gradient of acute temperature change, encompassing 17–41°C. 2) to measure the mRNA expression level of Hsp70 at the maximum (41°C) and minimum (17°C) temperature, in order to link the cellular stress level with the temperature-driven metabolic benchmarks. With this approach we intended to exemplify the utilization of an aerobic performance window delimited by critical -low and -high temperature challenge as tool to reliably determine energetic key parameters similar to the traditional SMR, MMR and AS for slow-moving marine invertebrate species. With the combined analysis of O_2_ consumption and Hsp70 gene expression we examine the synergy between these two key stress markers to comprehensively study functional disruption of homeostasis and prioritization of cellular protection mechanisms, due to thermal stress in *H*. *scabra* and other similar species.

## Materials and methods

### Study animals and experimental design

All experiments were conducted in accordance with the German ethics on animal welfare. This study was conducted at the Indonesian Institute of Science (LIPI), Lombok, Indonesia. In January 2016, a number of 32 juvenile *H*. *scabra* (63 ± 15g) were lawfully collected with a LIPI fishing permit from the lower intertidal zone off the West-Coast of Lombok, Pantai Sira di Pagi Hari (8°22´5.42´´S, 116°6´58.37´´E). After specimen collections, the animals were transported to the LIPI aquaculture facilities and placed in aquaculture tanks (100L), with flow-through conditions (29°C; 35ppt). Natural sediments were provided as food source. After an acclimation period of 14 days, four animals were placed over a 24h defecation period in a separate tank without sediment (50L; 29°C; 35ppt). Subsequently, four animals were randomly divided into two groups (n = 2) and placed into gas-tight Acrylic Chambers (AC; V = 600ml), which were submersed in two separate water baths (20L aerated and filtered seawater; 29°C; 35ppt). Each water batch contained two animals, after one hour acclimation to AC conditions, the temperature in one water bath was changed by 2°C per hour while the temperature in the other water bath was kept constant at 29°C (control). This experimental setup was separately repeated eight times (n = 16), with four experiments allocated to incremental temperature increase (29–41°C; n = 8) and four to incremental temperature decrease (29–17°C; n = 8). Right after the cold and warm temperature manipulation experiments the animals were anesthetized using 0.5% magnesium sulphate [27]. Subsequently, the respiratory tree (RT) was removed and immediately snap-frozen in liquid nitrogen for Hsp70 analysis.

### Temperature-induced changes in O_2_ consumption

**Fig 1:**
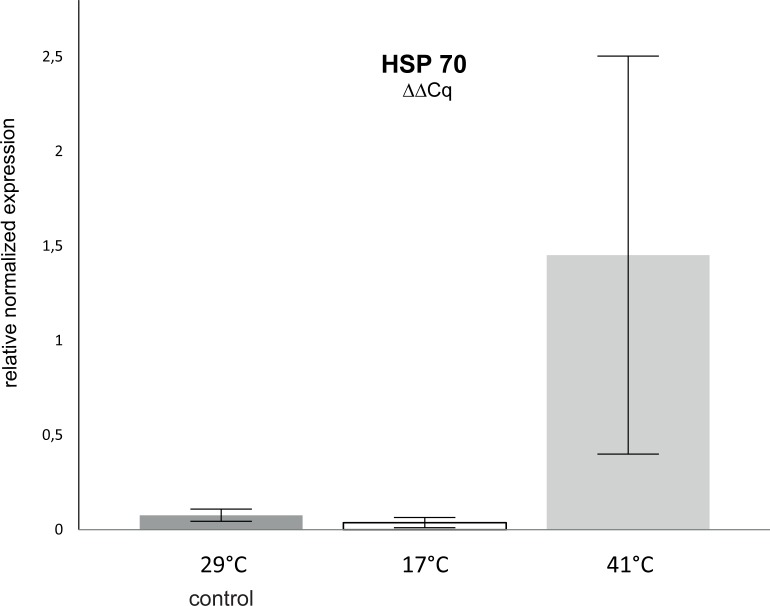
Average (mean ± standard deviation) relative normalized Hsp70 gene expression, for the two temperature manipulation experiments with eight biological replicates (n = 8), (1) cold (17°C; n = 8; p = 0.34) and (2) warm (41°C; n = 8; p = 0.00086) compared to the pooled controls (29°C; n = 16). Differences between treatments were calculated using a Mann-Whitney test. Significant differences are indicated for P<0.05 (*) and P<0.01 (**).

Intermitted-flow respirometry was used to determine resting- and maximum- O_2_ consumption for eight *H*. *scabra* individuals through incremental cold and warm temperature manipulations, respectively. Respirometry measurements were conducted always at the same daytime. Submersible pumps (150L h^-1^) provided a constant water supply from the water bath through the acrylic chamber (AC), while a peristaltic pump maintained internal water mixing within the AC. The water flow into the AC was stopped for 15min every 15min. This interval of continuous flushing cycles and the change rate of temperature (2°C h^-1^) enabled one respiration measurement cycle per degree of temperature up- and down- regulation: (1) acute temperature elevation (29°C, 31°C, 33°C, 35°C, 37°C, 39°C, 41°C); and (2) acute temperature decrease (29°C, 27°C, 25°C, 23°C, 21°C, 19°C, 17°C). At each temperature the water flow interruption time was short enough to ensure O_2_ concentrations above 80% saturation [17]. The temperature-compensated O_2_ concentration (mg L^-1^) over time (s), was continuously recorded (1 s^-1^) for each AC using oxygen-sensitive REDFLASH dye on contactless spots (2mm) glued at the inside of the AC lids and linked to a Firesting Optical Oxygen Meter (Pyro Science e. K., Aachen, Germany) via fibre-optic cables. For consistency, the first and last minute of the resulting respiration slopes were excluded from analyses. For each temperature change (2°C h^-1^), the average O_2_ consumption rate (μg g^-1^ h^-1^) was calculated from the respiration slopes (R^2^≥0.90) of the eight biological replicates (n = 8). To account for microbial background respiration rates the oxygen depletion in the empty AC´s, before the animals were placed in (pre-blank) and after the animals were removed (post-blank), was measured over 15min. The background O_2_ consumption rates were calculated for each temperature, assuming a linear microbial accumulation between pre-blank and post-blank, and subtracted from the respective animal respiration slope. Mean respiration rates (n = 8) were calculated per temperature. Within the window of linear correlation between temperature and O_2_ consumption, peak and minimum respiration was defined as temperature-induced maximum (TMMR) and standard (TSMR) metabolic rates at the corresponding critical warm (WT_crit_) and cold (CT_crit_) temperatures, respectively (see Fig 1). Mean TMMR and TSMR values served to determine a temperature-induced aerobic scope (TAS) (Eq 1). Along the entire temperature gradient, no clear pattern of activity changes were monitored for the animals. To account for temperature effects along the temperature gradients, the temperature quotients (Q_10_) were calculated using mean O_2_ consumption (R_1_ and R_2_) at control temperature (29°C; T_1_) and at WT_crit_ (T_2max_) and CT_crit_ (T_2min_) (Eq 2).

TAS=TMMR−TSMR (1)

$$Q_{10}={{(R}_{2}/R_{1})}^{(10/\left(T_{2}-T_{1})\right)}$$ (2)

### RNA extraction and cDNA synthesis

100mg deep-frozen respiratory tree (RT) samples were immediately homogenized in 1 ml of TRIzol Reagent (Life Technologies, California, USA). The RNA extraction followed the manufacturer recommendations. The quantity of the extracted RNA was measured in a BioPhotometer (Eppendorf, Hamburg, Germany). Possible contaminations of genomic DNA were removed using DNase (Promega, Madison, WI) digestion according to the manufacturer recommendations. Subsequently, single-stranded cDNA was synthesized at 42°C for 60min, using the GoScript^TM^ Reverse Transcriptase (Promega, Madison, WI) following the manufacturer recommendations. The reverse transcription was based on 4μl total RNA template, mixed with 0.5μl oligo(dT)_20_ and 0.5μl of random primers.

### Primer design for the RT-qPCR assay

Besides Hsp70, two established reference genes 18S rRNA (18S ribosomal RNA) and ACTB (*β*-actin) [28, 29] were targeted in this study. 18S sequences are available for six sister species of the genus *Holothuria* (AY133470.1 –AY133475.1). These published sequences were used to generate a homologous sequence alignment using BioEdit 7.2.5 [30] to identify conserved primer binding sites. Quality control of the primers was conducted using PCR Primer Stats [31]. *β*-actin and the target gene Hsp70 were not available for any *Holothuria* species. This gene was, therefore, targeted using RACE-PCR [32] with a degenerated forward primer in combination with an anchor-primer (introduced at the cDNA synthesis using an anchored OligoT primer (Odt7)). The *β*-actin forward primer (ACTB_FW: ACTCTGCTACGTCGCTCTTG) was designed from *Apostichopus japonicas*, whereas, Hsp70 followed the following approach: The known *A*. *japonicus* sequence (EU930813.1) was utilized to BLAST [33] the Sequence Read Archive (SRA) of *Holothuria glaberrima*. The best 50 hits were imported to the software BioEdit 7.2.5 followed by a contig assembly program [34]. Three out of seven contigs were selected for further alignments with homologous Hsp70 sequences from animals in various taxa: *Apostichopus japonicas* (EU930813.1), *Parastichopus californicus* (GAVO01016544.1, GAVO01019403.1), *Ciona intestinalis* (AK116745), *Lottia gigantea* hypothetical protein mRNA (XM_009047345), *Psammechinus miliaris* (FN796462) and *Brissopsis lyrifera* (FN667017). The function similarity matrix (for shading) was applied in the alignment window to detect conserved regions, from which the Hsp70 primers were designed.

The degenerated forward primer Hsp70_480_fw (CCRGAAGAAATYAGYTCSATGGT) was used in combination with the Odt7 anchor primer at 47°C and 35 cycles. 1μl of the resulting PCR product was used as template in a semi-nested PCR using Hsp70_680_rv (TTCTTATCGAGCCCATAGGC) at an annealing temperature of 47°C and 35 cycles. All PCRs used the Opti*Taq* DNA Polymerase kit (Roboklon, Germany) for the amplification. The PCR products of the expected size for Hsp70, 18S and β-actin were treated prior to Sanger sequencing using ExoSAP-IT PCR Product Cleanup kit (Affymetrix) to eliminate the unconsumed primers and nucleotides. After the identity of the PCR products was confirmed by Sanger sequencing (StarSEQ GmbH Germany), the resulting contig was used as a query sequence in a Primer-BLAST on NCBI for final qPCR primer design. [35]. Primer quality was analysed using PCR Primer Stats of the oligo analyser 3.1 software [36]. The chosen primer parameters were set to have a PCR product above 70 bp. The melting temperature fell into a range of 57°C to 63°C. The Echinodermata Refseq mRNA database was used to exclude amplification of wrong targets. The resulting *H*. *scabra* primers were tested at three different annealing temperatures (52°C, 54°C, 56°C) and 5% DMSO per PCR reaction was added to reduce primer dimer formation.

### RT-qPCR

The expression of Hsp70 was measured relative to the expression level of the two control genes 18S and β-actin in RT tissue. Measurements were conducted in three technical replicates using sterile H_2_O as negative control and a confirmed cDNA sample as positive control. Per sample, the final qPCR mixture contained 7.8μl sterile H_2_O (sterile DEPC-water, Roth), 10μl iTaq Universal SYBR Green Supermix (Bio-Rad), 0.1μl of the respective forward and reverse primers (HSP70, 18S, β-actin; 500nM in mix) (Biomers GmbH, Ulm, Germany) and 1.5μl cDNA sample. The qPCR reactions were conducted on the CFX Manager^TM^ Real Time PCR System (Bio-Rad Laboratories Inc., California, USA). The cycling protocol included an initial heating phase (95°C; 3 min), followed by 45 repeats of denaturation (95°C; 10s) and elongation (60°C; 30s), and finally the melting curve (95–60°C, 0.2°C s^-1^) to confirm single amplification products without primer dimers.

### Differential gene expression analysis

The output data (Cq-values) from the RT-qPCR measurements were exported from Bio-Rad CFX Manager (Version 3.0) software to Microsoft Excel 2010 to generate the bar plot. The expression levels of the target gene Hsp70 are given in calibrated normalized relative quantities (CNRQ values) ± the 95% confidence interval (CI), which automatically account for replicate variability, amplification efficiency and normalization factors. To test the stability of the two reference genes 18S and β-actin, geNorm [37] was utilized. Homogeneity of reference targets was assumed at M values <0.5. Mean (n = 8) relative Hsp70 expression levels of cold (17°C) and warm (41°C) treated animals were compared to the pooled (n = 16) Hsp70 expression levels of the animals maintained at control temperature (29°C). Differences were determined through a non-parametric Mann-Whitney test. Significant differences were assumed at P<0.05 (*) and highly significant differences at P<0.01 (**).

## Results

### Oxygen consumption

**Fig 2:**
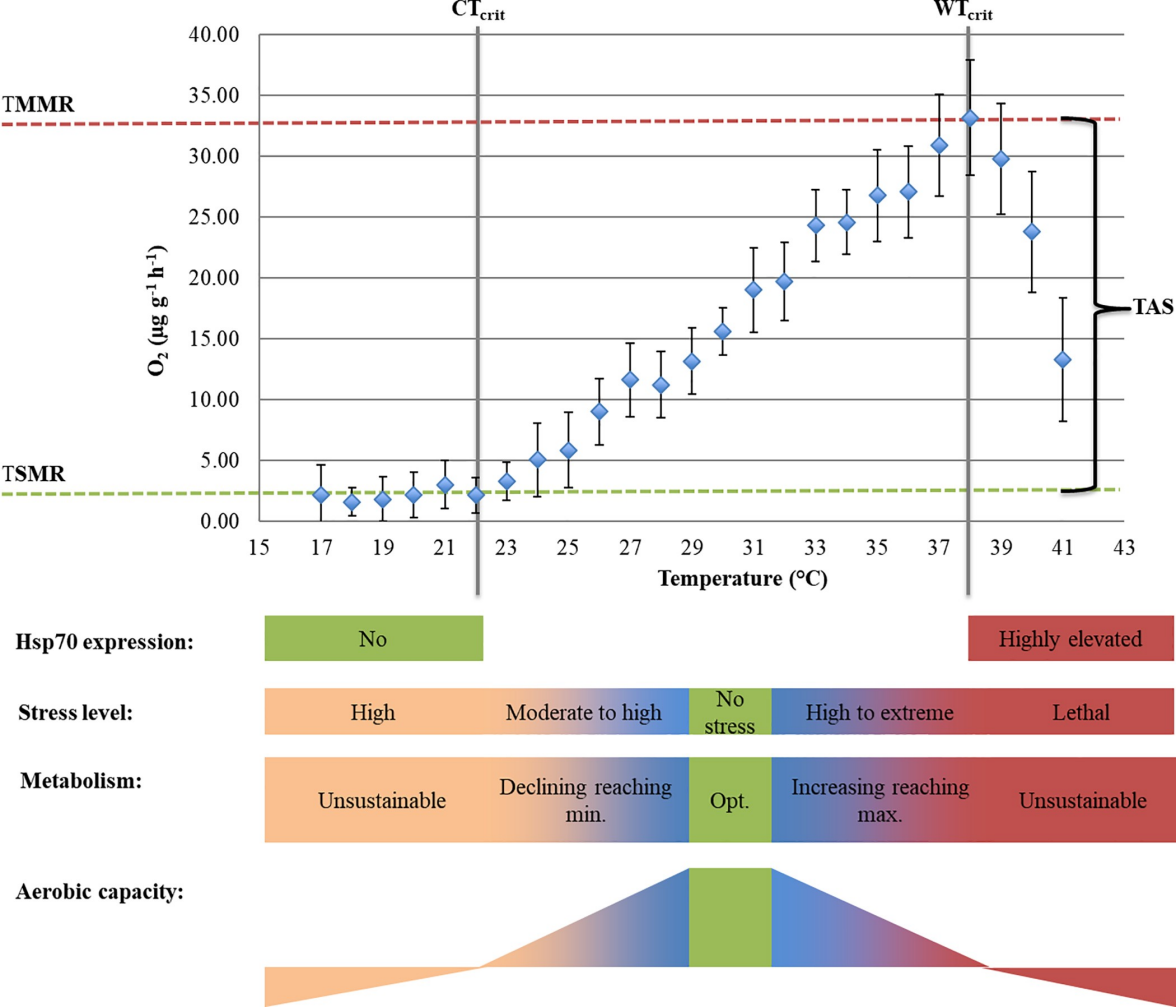
O_2_ consumption rate of *Holothuria scabra* at different temperatures, obtained from four warm and cold experimental replications. Points represent means ± s.d. of eight biological replicates (n = 8). Upper red dotted line indicate temperature-induced maximal metabolic rate (TMMR), at the critical warm temperature (WT_crit_; 38°C), lower red dotted line marks the start of the temperature-induced standard metabolic rate (TSMR), at critical cold temperature (CT_crit_; 22°C). The difference between TMMR and TSMR define the temperature-induced aerobic scope (TAS) for *H*. *scabra* (black brace). Beyond CT_crit_ and WT_crit_ (22–38°C) progressively negative aerobic performance caused by growing dependency on energy reserves and declining aerobic capacity, respectively, indicate homeostatic disruption. Overexpression of Hsp70 occurs only at the warm temperature maximum (41°C).

Within the range of 22–38°C, temperature and oxygen consumption rates (OCR) of *H*. *scabra* were significantly positively correlated (R^2^ = 0.99) (Fig 2). At 38°C the animals reached a temperature-induced maximal metabolic rate (TMMR) of 33.2 ± 4.7μgO_2_ g^-1^ h^-1^ (n = 8). At maximum temperature (41°C) OCR dropped to 13.3 ± 5.1μgO_2_ g^-1^ h^-1^ (n = 8). At22°C *H*. *scabra* reached its temperature-induced standard metabolic rate (TSMR) at 2.2 ± 1.4 μgO_2_ g^-1^ h^-1^ (n = 8). The animals maintained at constant ambient (control) conditions (29°C) exhibited a mean OCR of 13.2 ± 2.7μgO_2_ g^-1^ h^-1^ (n = 8). The difference between mean TMMR and TSMR revealed a temperature-induced aerobic scope (TAS) of 31μgO_2_ g^-1^ h^-1^ (see Table 1 and Fig 2). The temperature quotient (Q_10_) reached 13.2.7 for the OCR change between 22–29°C (temperature down-regulation) and 2.8 for the OCR change between 29–38°C (temperature up-regulation) Tables 1 and 2.

**Table 1:** O_2_ consumption rates at control conditions (T_cont =_ 29°C), critical cold temperature (CT_crit_ = 22°C) and critical warm temperature (WT_crit =_ 38°C). Temperature-induced standard (TSMR) and maximal metabolic rate (TMMR) indicate aerobic performance at CT_crit_ and WT_crit_, respectively. The temperature-induced aerobic scope (TAS) is given as difference between TMMR and TSMR. Temperature quotients (Q_10_) are given for the respective temperature gradients.

	Temp. (°C)	O_2_ consumption (μg g^-1^ h^-1^)	Q_10_	
			22–29°C	29–38°C
Control	29	13.2 ± 2.7		
TSMR	22 (CT_crit_)	2.2 ± 1.4	13.2	
TMMR	38 (WT_crit_)	33.2 ± 4.7		2.8
TAS		31		

**Table 2:** Primer sequences, for the three target genes (Hsp70, β-actin and 18S) in *H*. *scabra*, and technical details: Number of amplicon base pairs (bp), melting temperature (Tm) and total content of Gs and Cs (GC).

**Primer pair**	Sequence (5'->3')	Amplicon (bp)	Tm (°C)	GC (%)
Hsp70 (forward)	ATCCCGTTACCCATGCTGTG	145	60.11	55.00
Hsp70 (reverse)	AGCCCATAGGCAATAGCAGC		60.25	55.00
β-actin (forward)	ACTCTGCTACGTCGCTCTTG	143	58.7	55.0
β-actin (reverse)	GGAAGAGTGTCTCTGGGCAA		58.6	55.0
18S (forward)	GCTACTACCGATCGAATGGC	161	57.2	55.0
18S (reverse)	GATCCATCTGCAGGTTCACC		57.5	55.0

### Primer design

BLAST results confirmed the identity of control and target genes (ENA Acc. No. PRJEB25057 of published sequences) and Primer BLAST led to the successfully tested primer sequences listed in Table 2. All products were confirmed by sequencing.

### Hsp70 gene expression

The animals that were exposed to the acute temperature elevation (+12°C), up to 41°C within six hours, showed a highly significant up-regulation of Hsp70 (p = 0.000178) in the respiratory tree tissue, compared to the control animals kept at constant temperature (29°C) over the same time period. In contrast to that, a temperature decrease of the same magnitude (-12°C) did not affect Hsp70 expression in *H*. *scabra* (Fig 1 and Table 3). During both experiments the two reference genes, β-actin and 18S, exhibited stable homogeneityexpression.

**Table 3:** Hsp70 gene expression levels shown in mean calibrated normalized relative quantities (CNRQ) ± 95% confidence interval (CI), for both temperature manipulation experiments. Significant differences are indicated for P<0.05 (*) and P<0.01 (**).

**Heat shock**					
Gene	Temperature (°C)	Mean (CNRQ)	+ 95% CI	- 95% CI	P-value
Hsp70	41°C	3.24	7.77	1.35	2.81E-5**
	29°C (control)	0.15	0.3	0.07	
**Cold shock**					
Hsp70	17°C	1.09	3.04	0.39	0.75
	29°C (control)	0.91	2.19	0.38	

## Discussion

### Definition of TSMR, TMMR and TAS

The aerobic scope (AS) is widely considered as an important measure to determine the response of marine species to future ocean warming scenarios [9, 10]. The quantification of aerobic scope requires the accurate and species-specific quantification of standard metabolic rate (SMR) and maximal metabolic rate (MMR). In this study, we modified the traditional concept of physically induced SMR, MMR and AS by defining a temperature-induced standard metabolic rate (TSMR), maximal metabolic rate (TMMR) and aerobic scope (TAS) as suitable alternative metrics for the sea cucumber *Holothuria scabra* (representative of a slow moving, bottom-dwelling marine invertebrate species).

During acute temperature stress, the energy demand for basal maintenance increases with warming, and decreases with progressively colder temperatures. Basal maintenance is, however, limited by a maximal- and minimal- metabolic capacity (e.g. oxygen uptake- or mitochondrial- efficiency) [38]. Hence, the aim of this study was to expose *H*. *scabra* to acute temperature change (2°C h^-1^; 17–41°C) until critical cold (CT_crit_) and critical warm (WT_crit_) temperatures caused bottom (TSMR)- and peak (TMMR)- O_2_ consumption rates, respectively. Benthic intertidal ecosystems exhibit a high degree of thermal heterogeneity. Driven by water level changes, differences in topography, substratum type and vegetation, drastic temperature gradients can occur over very short time periods and spatial distances [39]. *H*. *scabra* is a tropical intertidal species hence, the definition of one overarching ecological relevant temperature change rate for this sedentary invertebrate was inexpedient. Instead of attempting a precisely simulated profile of natural temperature ramping, our goal was to generate comparable thermal tolerance estimations [5, 40, 41] A temperature ramping of 2°C h^-1^ was widely used to investigate the thermal tolerance of tropical [5, 41] and temperate [42] intertidal invertebrates. A similar rate of temperature change (1–4°C h^-1^) was also applied in other key studies investigating the role of oxygen limitations on the thermal tolerance of ectotherms [43–48]. Previous studies stressed the link between the rate of temperature ramping itself on maximal thermal tolerance levels [49–51]. Consequently, the thermal limits provided in this study must be tightly linked to the utilized ramping protocol and should not serve as general temperature threshold levels for *H*. *scabra*.

*H*. *scabra* exhibited TMMR (33.2 ± 4.7 μg g^-1^ h^-1^) at a WT_crit_ of 38°C. TSMR (2.2 ± 1.4 μg g^-1^ h^-1^) was defined as the start of a metabolic stable state condition, which was reached at a CT_crit_ of 22°C. Following the definition of the traditional AS the TAS (31 μg g^-1^ h^-1^) was calculated based on the difference between the means of the proposed alternative metrics TMMR and TSMR. Above critical temperatures, animals rely progressively on unsustainable anaerobic energy supply [14]. Accordingly, *H*. *scabra* showed declining respiration above WT_crit_, although the intensity of temperature stress increased, as may be indicated by the significantly elevated mRNA expression of Hsp70 at the peak temperature (41°C). Thus, at temperatures between 39–41°C we expect animal survival only over a very short time span (Fig 2). Critical cold temperature mark the point at which the temperature-induced metabolic rate matches the minimal energy requirements to sustain life functions. This basal maintenance cost is equivalent to SMR [38, 52]. In the present study, *H*. *scabra* exhibited the initiation of metabolic stable-state conditions at CT_crit_ (22°C). At temperatures (17–21°C) below this critical point, *H*. *scabra* presumably suffered from a fading energy balance to sustain minimal basal requirements, where any expenses for basal maintenance depend progressively on energy depots. Besides a growing energetic effort to sustain life functions below CT_crit_, the molecular analysis revealed no damaging cold temperature effects in form of Hsp70 overexpression at 17°C (Figs 1 and 2).

The presented temperature dependent aerobic window, delimited by TMMR, TSMR and TAS, may provide reliable metrics for slow-moving species like *H*. *scabra* to determine energy-homeostasis, and to distinguish between ecologically and physiologically important states similar to the traditional AS [53]. A common approach is to define species-specific thermal optima as those temperatures at which the AS peaks. Whereby, SMR and MMR are physically induced at several points to quantify AS changes along a temperature gradient. In this study, however, a temperature gradient itself was used to determine TMMR and TSMR, revealing a single TAS value (31 μgO_2_ g^-1^ h^-1^), which is linked to the control temperature (29°C) in this study. Future studies should determine TMMR and TSMR for different acclimation temperatures, this would allow a true comparison between AS and TAS in terms of thermal optima detection. Besides the primary goal to provide an alternative to the traditional AS concept, the presented aerobic window, delimited by TMMR, TSMR and TAS, allows unique insights into temperature dependent aerobic capacity of *H*. *scabra*. Dividing the TAS by two reveals a metabolic rate (18.7 μgO_2_ g^-1^ h^-1^) in the exact center between TMMR and TSMR. Consequently, the temperature (31.5°C) that corresponds to this central metabolic rate indicates a point at which equal aerobic resources towards cold and warm temperature stress are available. This specific temperature may serve to define a form of thermal optimum. For some reef fish the highest aerobic scope was found at temperatures of 1–2°C above summer maxima values [6]. The defined optimal temperature of 31.5°C based on TAS, exceeds the ambient natural conditions at our study site (max. 30°C; personal communication with sea cucumber farmers) by a similar magnitude. In previous studies, the oxygen consumption rate of *H*. *scabra*, at unstressed conditions, ranged from 9–12μgO_2_ g^-1^ h^-1^ [54] and 11–16 μgO_2_ g^-1^ h^-1^ [55]. In this study we assume unstressed conditions between control temperature (29°C) and the defined thermal optimum (31.5°C), the corresponding optimal performance range of 13.85–18.7 μgO_2_ g^-1^ h^-1^ exceeds this literature values only slightly at the upper end.

Another difference between the traditional AS and the TAS is that in contrast to metabolic change induced by physical challenge, temperature challenge causes thermally induced adjustments of the metabolic system, but also through the effect of temperature itself. The rate of metabolic change, solely as response to temperature, is predictable through the temperature quotient Q_10_ (approximately a doubling or tripling of a given rate function for every 10°C of temperature increase, Q_10_≈2–3) [56, 57]. *H*. *scabra* was able to adjust its aerobic performance as response to acute temperature change within the range of 22–38°C. Within the linear phase of the temperature up-regulation experiment (29–38°C), *H*. *scabra* exhibited a Q_10_ of 2.8 (Table 1), which is within the expected range. Along the linear regression during temperature down-regulation (22–29°C), however, the Q_10_ value was dramatically higher (Q_10_ = 13.2) (Table 1). This gives a clear evidence of a strongly pronounced metabolic rate response due to cold stress. Interestingly, the sharpest decline in O_2_ consumption occurs below 24°C (5.1 to 2.2 μgO_2_ g^-1^ h^-1^), temperatures below 25°C were also defined in other studies as point at which overall activity and feeding rate declined distinctly in *H*. *scabra* [58, 59]. In long-term exposure experiments over 30 days, however, *H*. *scabra* was able to successfully acclimate to water temperatures of 33°C and 21°C, by adjusting respiration rate and metabolic enzyme activity [60]. These results show that *H*. *scabra* survived and remained active over a prolonged period at temperatures even below 22°C. This matches with the present results, which predict the optimal performance of *H*. *scabra* up to relatively high temperatures (31.5°C) and no induction of cellular stress (Hsp70) at temperatures down to 17°C. The temperature tolerance of *H*. *scabra* might be linked to life stages and specific habitat preferences. The studied animals represent intermediate sized juveniles, collected solely from the intertidal zone. This means that the relatively wide temperature tolerance exhibited by the studied *H*. *scabra* may not represent the thermal tolerance of larger animals found in the subtidal zone.

### mRNA expression of Hsp70

Hsp70 is a highly conserved cellular chaperon and known as universal stress response in many taxa, ranging from bacteria via plants to mammals [61, 62]. To date, Hsp70 sequences were only available for two sea cucumber species: *A*. *japonicas* [63] and *H*. *tubulosa* [64]. In this study, we successfully targeted the full-length cDNA sequences of Hsp70, 18S and β-actin in respiratory tree tissue of *H*. *scabra*. Above 38°C Hsp70 gene expression was highly elevated in *H*. *scabra*, although whole-organism respiration decreased sharply. This provided evidence that Hsp70 is intensively involved in cellular defence mechanisms at those temperatures indicating the homeostatic disruption in *H*. *scabra* between 39–41°C. Negative effects such as reduced cell growth rates [65] and productivity [66] can be associated with enhanced Hsp70 expression. This interaction is in line with our findings, which showed a decline in respiration, while Hsp70 was highly expressed.

The temperate sea cucumber species *Apostichopus japonicus* showed significantly up-regulated Hsp70 levels at an acute temperature increase up to 20°C and 25°C, compared to 16°C (control) where the highest Hsp70 expression corresponded to the highest temperature [59]. For the tropical gastropod species *Pomacea canaliculata*, the induction temperature for Hsp70 expression was at 36°C, whereas maximal Hsp70 expression was reached at 42°C. Cold temperature on the other hand caused only slight reductions in Hsp70 expression at temperatures below 16°C [67]. This temperature range for *P*. *canaliculata* is in line with our findings for *H*. *scabra*, which revealed a highly significant up-regulation of Hsp70 after a fast temperature increase, up to 41°C, but no significant effect after a fast drop in temperature, down to 17°C. Along a similar temperature gradient (20–41°C) the freshwater clam *Corbicula fluminea* exhibited overexpression of Hsp70 at elevated temperature with protein synthesis starting at 38°C, whereby the heat shock response was prioritized over the oxidative stress response [68]. Similarly, the clear respiration peak of *H*. *scabra* at 38°C in this study mark a critical point at which the capacity limits of oxygen supply were reached, marking the onset of metabolic depression and resource allocation towards the heat shock response. Although the Hsp70 induction temperature was not defined in this study, the pattern of stable Hsp70 expression at lower temperatures, and the significant Hsp70 up-regulation at warm conditions, show clear similarities between *P*. *canaliculata*, *C*. *fluminea* and *H*. *scabra*. Similar expression pattern were also found for the insect *Drosophila melanogaster* [69], and the two fish species *Danio rerio* [70] and *Oncorhynchuss mykiss* [71]. For cold resistance, Hsp70 seems to play a minor role [72]. Acute cold-stress is not affecting secondary and tertiary protein structures to the same extent as heat-stress. Thus, cellular chaperones are not immediately required. Long-term cold acclimation, however, has shown to induce elevated Hsp70 levels, which was associated with the occurrence of chilling injuries [73]. In the present study, those *H*. *scabra* that were exposed to 17°C showed no differences in Hsp70 expression levels, compared to control animals (29°C). This indicates that a water temperature of 17°C was still above the threshold level at which cellular protection mechanisms were initiated, although aerobic performance was negative.

## Conclusions

This study defined a temperature dependent aerobic window, delimited through critical cold (CT_crit_) and critical warm (WT_crit_) temperatures at which O_2_ consumption levelled (TSMR) and peaked (TMMR), respectively, for the sea cucumber *Holothuria scabra*. The identified metrics proved to be practical to calculate a temperature-induced aerobic scope (TAS), which may serve as suitable alternatives to the traditional aerobic scope (AS) for *H*. *scabra* and other sluggish bottom-dwelling species alike. To fully explore the potential of the presented approach, however, TMMR, TSMR and TAS need to be quantified at different ambient temperatures and during other stress exposures, to enable detailed comparisons to traditional AS analyses. Future studies should verify whether the TAS shrinks and expands in a similar pattern as AS, due to changes in baseline conditions such as acclimation temperature. In this study the acute temperature induction, to identify TMMR and TSMR, by itself revealed an extraordinary thermal tolerance of *H*. *scabra*, especially towards acute warming. These findings provide evidence that *H*. *scabra* is capable to endure extreme temperature events beyond current predictions of the IPCC [74], at least over the limited time span presented in this work.

The established primers for the targeted Hsp70 gene and the two control genes β-actin and 18S led to a reliable qPCR output. These data provide the first insights into Hsp70 differential gene expression in *H*. *scabra* at critical cold (17°C) and warm (41°C) temperatures. While Hsp70 was highly overexpressed at 41°C no increased expression was measured at 17°C. In combination with the identified TMMR and TSMR, highly elevated Hsp70 expression could be linked to a clear decline in respiration above 38°C) while no Hsp70 upregulation was observed at the respiratory base line below 22°C. The quantification of Hsp70 gene expression and definition of the aerobic benchmarks TMMR, TSMR and TAS showed promising synergies for thermal stress detection in *H*. *scabra*. Defining WT_crit_ as critical temperature point beyond which a surpassed mitochondrial capacity and the onset of metabolic depression causes declining oxygen uptake, the increased expression of Hsp70 indicates a transition towards induction of the cellular heat shock response (HSR). While above WT_crit_ the synergy of raised HSR and falling respiration may imply acute homeostatic disruption and lethal thermal stress, the combination of base budget O_2_ consumption and no upregulated Hsp70 expression below CT_crit_ the suggests unsustainable energetic expenses to sustain basal maintenance, but no yet detrimental cold stress. These interpretations rely on the assumption that Hsp70 expression in *H*. *scabra* was not impaired by cold temperature itself and that the expression level did not peak earlier, during the temperature manipulations, before the lowest and highest temperature were reached. The integration of molecular expression patterns into physiological analyses add important knowledge to enhance the understanding of energy resources allocation at the onset of aerobic depression to fuel cellular defense mechanisms.

Future studies should compare Hsp70 expression levels in *H*. *scabra* at more temperature endpoints and exposure times, utilizing our suggested primers to define the exact Hsp70 induction points for this species. Overall, this study explored a novel approach for fine-tuned thermal stress detection in *H*. *scabra*, which may help to optimize culture conditions and to predict future ocean-warming effects for this species and beyond.
